# New Insights into Molecular Mechanism behind Anti-Cancer Activities of Lycopene

**DOI:** 10.3390/molecules26133888

**Published:** 2021-06-25

**Authors:** Boon-Peng Puah, Juriyati Jalil, Ali Attiq, Yusof Kamisah

**Affiliations:** 1 Drug and Herbal Research Centre, Faculty of Pharmacy, Universiti Kebangsaan Malaysia, Jalan Raja Muda Abdul Aziz, Kuala Lumpur 50300, Malaysia; p102604@siswa.ukm.edu.my; 2 Faculty of Pharmacy, MAHSA University, Bandar Saujana Putra, Jenjarom 42610, Malaysia; aliattiq@mahsa.edu.my; 3 Department of Pharmacology, Faculty of Medicine, Universiti Kebangsaan Malaysia, Cheras, Kuala Lumpur 56000, Malaysia; kamisah_y@ppukm.ukm.edu.my

**Keywords:** lycopene, tomatoes, cancer, immune system, inflammation

## Abstract

Lycopene is a well-known compound found commonly in tomatoes which brings wide range of health benefits against cardiovascular diseases and cancers. From an anti-cancer perspective, lycopene is often associated with reduced risk of prostate cancer and people often look for it as a dietary supplement which may help to prevent cancer. Previous scientific evidence exhibited that the anti-cancer activity of lycopene relies on its ability to suppress oncogene expressions and induce proapoptotic pathways. To further explore the real potential of lycopene in cancer prevention, this review discusses the new insights and perspectives on the anti-cancer activities of lycopene which could help to drive new direction for research. The relationship between inflammation and cancer is being highlighted, whereby lycopene suppresses cancer via resolution of inflammation are also discussed herein. The immune system was found to be a part of the anti-cancer system of lycopene as it modulates immune cells to suppress tumor growth and progression. Lycopene, which is under the family of carotenoids, was found to play special role in suppressing lung cancer.

## 1. Introduction

Cancer originates from the dysregulation of vital genes involved in the cell maintenance system. This results in abnormal cell proliferation due to the imbalance between proto-oncogene and tumor suppressive gene [1]. There were more than 277 different types of cancers with different pathogenesis pathways, with genetic inheritance being the dominant factor of cancer development and progression [1]. The available effective treatments for cancer are generally chemotherapy and radiotherapy based. Chemotherapy is a type of standard cancer therapy with the use of drugs (bleomycin and cisplatin) with severe side effects such as vomiting for approximately 12 times per day after treatment [2]. Radiotherapy is a therapy which uses radiation to damage the DNA of cancerous cells, with the major limitation being the lack of specificity and thus, causing damage to the normal cells surrounding the malignant tumor. It can also lead to many side effects with respective to the types of underlying cancers such as nausea, vomiting and gastritis for liver cancer; bowel incontinence and rectal irritation for colorectal cancer; shortness of breath and radiation pneumonitis for lung cancer [3]. Though being known to be effective against cancer, they will eventually take their tolls on patients’ bodies, both mentally and physically. In the search of alternatives, some would turn to nutritional approach for cancer with the use of plant-based food which contains various nutrients and phytochemicals which could be helpful towards cancer.

**Figure 1:**
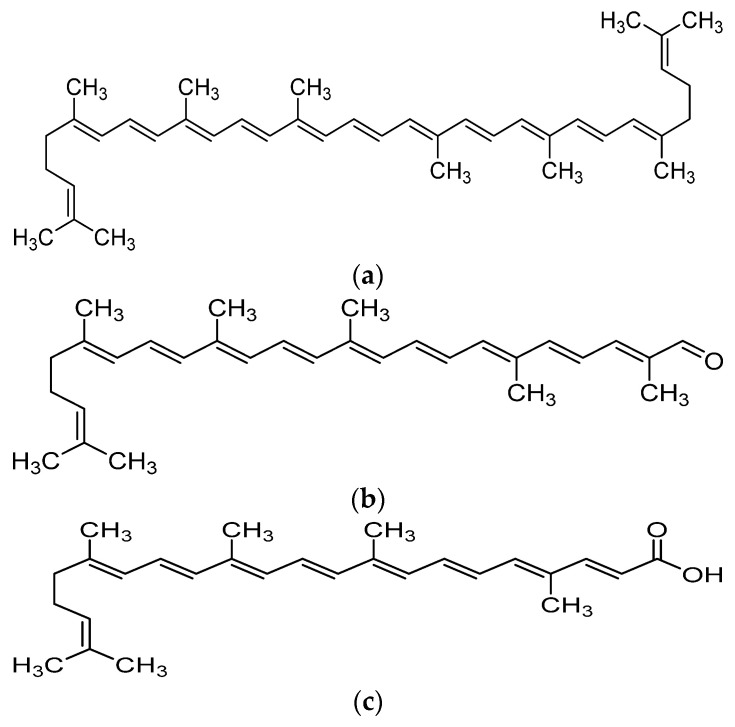
Chemical structure of lycopene and its metabolites with reported anti-cancer property. (**a**) Lycopene, (**b**) Apo-8′-lycopenal, (**c**) Apo-10′-lycopenoic acid.

Recent advances in the field of nutrition showed that bioactive compounds from plant-based food such as rutin, curcumins, tocotrienols could enhance the efficacy of chemotherapy and alleviate the side effects of chemotherapeutic drugs [4,5,6,7,8]. Naturally occurring carotenoids were also found to be associated with decreased risk of various cancer such as prostate breast, colorectal and ovarian cancer, mostly due to their antioxidant effect and effect in reducing carcinogenesis and regulating pathway which involves cell death and growth [9]. One of the bioactive compounds which was reported to possess anti-cancer activity was lycopene [10]. Lycopene is a carotenoid found commonly in fruits and vegetables such as tomatoes, pink grapefruit and watermelons with non-provitamin A activity. It is the compound responsible for the red coloration of the fruits. It shares the same molecular mass and chemical formula with beta-carotene, but lycopene is an open-polyene chain which lacks the β-ionone ring structure found in beta-carotene. Lycopene is a highly unsaturated hydrocarbon which is able to undergo cis-trans isomerization under induction by light, thermal or chemical reactions. Most of the lycopene found in nature, from existing plant are predominantly in trans-configuration, which is more thermodynamically stable than its cis-counterpart [11,12]. Years of research managed to identify some of the metabolites of lycopene found in human body, which could depict an idea of the metabolism of lycopene in human. Some of the known metabolites of lycopene which were detected in plasma of humans include apo-8′-lycopenal, apo-10′-lycopenal, apo-10′-lycopenoic acid and apo-10′-lycopenol [13]. Apo-8′-lycopenal and apo-10′-lycopenoic acid were the metabolites found to possess anti-cancer activity, despite being a degraded fragments of lycopene [14,15] (Figure 1). Such observation could reflect the potential of lycopene in anti-cancer as its bioactivity is considered robust as metabolic catabolism would not nullify its effect.

Lycopene was known to be able to suppress cancerous cell proliferation, migration, invasion and adhesion activity in cell culture studies. Such suppression was often observed with changes of cancer-related gene expression and relief of oxidative stress. In general, lycopene could suppress the expression of MMP-2, MMP-7, MMP-9, Sp1, IGF-1R, VEGF while increasing E-cadherin stabilization, connexin 43, nm23-H1, TIMP-1 and TIMP-2 levels [15,16,17,18,19,20,21,22]. One of pathways involved in the anti-cancer property exhibited by lycopene was associated with its ability to regulate apoptosis-related protein and gene expression such as caspase-3, caspase-8, Bax levels and Bax:Bcl-2 and Bcl-xL among cancerous cells [23,24,25,26]. However, over the years, there had been evidences showing that lycopene could exert anti-cancer activity via different molecular mechanisms than those mentioned above. Hence, in this review, we discuss the new found insights and perspectives of the anti-cancer activities possessed by lycopene. As compared to other comprehensive reviews about lycopene, we focus on discussing how the anti-inflammatory effects exhibited by lycopene could be related to its anti-cancer activity, how lycopene modulates the immune system to fight cancer and the selectiveness of lycopene in preventing lung cancer. By such discussion, we look forward to opening new outlooks for research and promote the importance of lycopene consumption as part of a healthy diet.

## 2. Metabolism and Bioavailability of Lycopene

The metabolism of lycopene is a complicated process, whereby it has to be released from the food matrix, emulsified and solubilized into micelles before absorption could occur as it is lipid soluble. Absorption of lycopene could occur either by passive diffusion or via SR-B1 transporter and CD36 surface membrane glycoprotein found in the small intestine. The absorption process is tightly regulated by intestine-specific homeobox (ISX) transcription factor and dependent on both intestinal β-carotene 15,15′-oxygenase (BCO1) and SR-B1 expression. After lycopene uptake by the small intestine, it will undergo isomerization from all-trans configuration to 5-cis lycopene and 13-cis lycopene, to be cleaved by carotene-9′,10′ monooxygenase (BCO2) to produce apo-10′-lycopenal. Apo-10′-lycopenal would then either be oxidized to apo-10′-lycopenoic acid or reduced to apo-10′-lycopenol. The metabolites mentioned above would later be packaged into chylomicrons and transported to the lymphatic system, liver and other peripheral tissues [27].

There are several factors which were found to be able to affect the bioavailability of lycopene from food. The release of lycopene from the plant itself is one of the determining factors of bioavailability while significant food processing also did find to improve the bioavailability of lycopene. It was proposed that the act of maceration could break down the plant cell walls and thus, leading to weakening of the bond between lycopene and the plant cell tissue matrix [28]. Some research suggested thermal processing could cause isomerization of naturally occurring all trans lycopene to cis lycopene, which is more easily oxidized and bioavailable towards humans [29]. Due to the fact that lycopene is highly lipophilic, consumption of lycopene with a certain amount of fats would greatly increase uptake rather than plain consumption as oil may improve absorption of lycopene by tissues [30]. However, it is recommended to avoid consumption of lycopene concurrently with high dietary fiber intake as several types of dietary fiber were found to be able to reduce the bioavailability of lycopene [31].

## 3. Role of Inflammation in Cancer Development and Progression

Inflammation itself is defined as a state whereby the immune system of our bodies has been invoked to heal the damaged parts and or remove external invaders from our bodies. It could either be acute or chronic, depending on the nature of the inducers. The link between inflammation and cancer was established based on the role of chronic inflammation in tumorigenesis. In theory, there are generally two pathways which could describe how inflammation and cancer are related, named intrinsic and extrinsic pathway. In the intrinsic pathway, neoplasia caused by accumulated genetic error would eventually lead to the existence of the microenvironment by upregulation of inflammation. In the extrinsic pathway, the existence of an inflammatory environment initiates the development of cancer [32].

It is a sequential development process which is initiated by chronic inflammation. Chronic inflammation is often associated with prolonged tissue damage, followed by damage-induced cellular proliferation and tissue repair as part of the healing process. The difference between chronic and acute inflammation is that such a process happened repeatedly in chronic inflammation, leaving the body in a prolonged state of inflammation. In chronic inflammation, metaplasia occurs due to the exposure of harsh environmental stimuli as a way for our bodies to adapt to the change and heal. Metaplasia is often being known as a precursor to low grade dysplasia, which could evolve to high grade dysplasia which is irreversible and eventually to carcinoma [33,34].

In inflammation, cytokines produced by immune cells such as macrophages and T cells are originally supposed to aid in the battle against invaders, by causing damage to the invaders. However, during the battle, damage inducing compounds such as free radicals could also cause significant damage to the nearby cells, leading to the incident of DNA damage. Chronic inflammation causes repeated damage to those cells, whereby accumulation of such damage eventually leads to the formation of mutation in proliferating epithelial cells [35]. Over the years, scientific evidence pointed out that proinflammatory cytokines might play roles in cancer progression as they are mostly inducible by hypoxia which tumor cells often face [36]. For instance, tumor necrosis factor (TNF) is a proinflammatory cytokine which promotes inflammation induced carcinogenesis, due to its ability to stimulate NF-κB and JNK signaling pathway. It could either be pro tumorigenic or anti tumorigenic depending on the pathways activated as NF-κB often promotes antiapoptotic effect while JNK promotes cell death [37]. IL-6 is another commonly known proinflammatory cytokines which are found in high levels in tumor microenvironment. It could modulate apoptosis, survival, proliferation, angiogenesis, invasiveness and metastasis which eventually lead to promotion of tumorigenesis. IL-6 also does offer DNA damage and oxidative stress protection towards cancerous cells by facilitating repair and induction of countersignaling pathways [38]. IL-1 was also found to be associated with development of metastasis as proven in mouse models whereby mice with IL-1 receptor antagonist suffered less tumor development [39]. Apart from proinflammatory cytokines, chemokines which play critical role in the recruitment of leukocytes to the inflammation sites such as IL-8 was found to be able to proliferate and encourage metastasis of melanoma cells [40].

## 4. Cancer Suppression through Immunomodulation

In general, the immune system eliminates cancerous cells from our body via both innate and adaptive immunity. Initially, inflammation caused by cancerous cells initiates both an innate and adaptive immune response which aims to resolve such inflammation as part of our body’s healing system [41,42]. Tumor cells usually could be recognized by our immune system via the expression of tumor associated antigen due to changes in their cell surface proteins. These antigens will be recognized by complement proteins and thereby exposing them to complement protein mediated death [43]. The second attacker which would pick up these changes in cell surface proteins would usually be NK cells. One of the remarkable changes in cell surface protein in cancerous cells would be its altered or reduced MHC class I expression cell surface marker [44]. Such abnormality would activate NK cells via binding of NK cell surface receptors to a surface glycoprotein on tumors. NK cells would then execute apoptosis via the release of cytoplasmic granules containing perforin and granzymes which later forms pores in cell membranes. NK cells could also release cytokines which activate antigen-presenting cells such as dendritic cells to build a link between innate immunity and adaptive immunity [44].

In adaptive immunity, the process would be more complicated as dominant lymphocytes rely on the activation of signals by antigen presenting cells. Due to their high specificity, effector cells such as T cells and B cells require stimulation of antigen presenting cells which had phagocytosed and processed the tumor associated antigen. These antigen presenting cells would have to load these antigens onto their MHC class II molecules which present exogenous peptides of tumor antigens and MHC class I molecules which present endogenous peptides of cancer antigens [45]. Presentation of these antigens to the receptors of CD4^+^ or CD8^+^ T cells would lead to activation and formation of memory T cells [41,46]. Secretion of cytokines such as IL-2 would encourage T cell proliferation [47] while activation of CD8^+^ T cells would initiate the induction of cell-mediated lysis of cancerous cells.

B cells activation often requires the presence of activated CD4^+^ T cells. In general, B cells can be activated either by thymus dependent or thymus independent mechanisms. Activation of B cells by CD4^+^ T cells with specific tumor antigen is commonly being known as thymus dependent activation. In thymus independent activation, B cells are activated by antigens themselves with highly repetitive structures [48]. After activation, B cells would start secreting antibodies which can lead to tumor cell lysis by either antibody dependent complement cytotoxicity or complement dependent cytotoxicity [49]. Tumor cell lysis could also occur as a result of antibody binding to Fc receptors on NK cells [50].

## 5. Anti-Inflammatory Activities of Lycopene

Many studies provided evidence whereby lycopene could suppress proinflammatory cytokines such as IL-12, TNF-α, IL-1, IL-1β, IL-6 [51,52,53,54] and it was suggested that the main mechanism behind the anti-inflammatory property exerted by lycopene was downregulation of proinflammatory cytokines and thereby preventing the promotion of inflammation. Inflammatory mediators were also targets of lycopene as lycopene was able to suppress the production of COX-2, PGE_2_, NO, iNOS under stimulation of LPS [55,56]. COX-2 or cyclooxygenase 2 is an enzyme which catalyzes the conversion of arachidonic acid to prostaglandins H_2_ and it is the major source of prostaglandins found in inflammation. PGE_2_ is being known as one of the prostaglandins which is highly regulated during inflammation and it serves as a mediator for several biological functions. It is known to be involved in inflammation as it involves in processes which contribute to a common sign of inflammation such as redness, pain and swelling [8,57,58]. Downregulation of COX-2 would eventually reduce the production of prostaglandins, leading to a well-controlled inflammatory state instead of excessive inflammation. Nitric oxide or NO is a signaling molecule that involves blood vessel relaxation and vascular tone regulation. It plays a role in the pathogenesis of inflammation as well as it could also function as an inhibitor for leukocyte adhesion to vascular endothelium for extravasation. NO is a byproduct produced by enzymatic reaction of L-arginine to citrulline as catalyzed by an enzyme named nitric oxide synthase (NOS). As an inflammatory mediator, the concentration of NO will decide its nature of being anti-inflammatory or proinflammatory. Under normal physiological circumstances, NO tends to be anti-inflammatory as it inhibits leukocyte adhesion to endothelium. However, in the case where NO tends to be proinflammatory, iNOS would be expressed among monocytes and other immune cells once there is the presence of proinflammatory cytokines. This will lead to high production of NO, which in this case, helps host defense by causing DNA damage towards the pathogen. NO was also produced by phagocytes during phagocytosis and this leads to a positive feedback loop whereby more NO would be produced to modulate the chemotaxis of monocytes to the inflammation site [59].

### Utilizing Anti-Inflammatory Activity of Lycopene against Cancers

The earliest evidence of lycopene being able to exert anti-cancer properties via inflammation resolution came out in 2006. In this study using colon carcinogenesis Sprague Dawley rat model, lycopene treatment after induction by azoxymethane caused suppression of aberrant crypt foci, preneoplastic lesion and biomarkers such as COX-2 and iNOS expression [60]. In MNNG gastric cancer rat model, administration of 50, 100 or 150 mg/kg body weight of lycopene caused an increment in antioxidant enzymes as expected (SOD, CAT, GSH-Px) and increment in cytokines (IL-2, IL-4, IL-10, TNF-α) and antibodies (IgG, IgA, IgM). Noticeable reduction in MDA and IL-6 were detected under the same study. The author suggested that the upregulation of both antioxidant status and immune system might play roles in the anti-cancer activity of lycopene [61]. In BCO2-knockout male mice, lycopene supplementation for 24 weeks in combination with high fat diet resulted in increment in hepatic lycopene, miR-199a/b, miR214 and decrement in hepatocellular carcinoma incidence, multiplicity, endoplasmic reticulum stress-mediated unfolded protein response, ER(UPR) and oncogenic biomarkers such as Met mRNA, β-catenin and mTOR1. With the same experimental design but in wild type mice, lycopene significantly exhibited anti-cancer property, mainly by inhibition of proinflammatory signaling as seen in significant reduction in phosphorylation of NF-κB p65, STAT3, IL-6 and suppressed inflammatory foci. In wild types mice, the author suggested that the anti-cancer property observed was mainly caused by the suppression and resolution of inflammation [62].

In cancerous cells, the anti-inflammatory property of lycopene could also be observed whereby it helped in alleviating some of the proinflammatory cytokines and biomarkers. In SW480 human colorectal cancer cells, lycopene treatment managed to reduce the gene expression of TNF-α, IL-1β, IL-6, iNOS, COX-2, PGE_2_ and NO via reduced NF-κB transcription and JNK signaling pathway inhibition [55]. The anti-inflammatory effect of lycopene was further explored in LNCaP, PC3 and DU145 prostate cancer cells and incubation with lycopene effectively reduced proinflammatory cytokines including IL-1, IL-6, IL-8 and TNF-α. A strong apoptotic effect was also observed in cancer cells. Similar experiment was done in vivo by utilizing tumor injection of prostate cancer xenografts. Mice which received lycopene administration had higher survival rate and reduced tumor burden [51]. Such anti-cancer evidence sheds light on the possibility of lycopene to modulate cancerous cell activity via alleviation of inflammation.

The anti-cancer activity of apo-10′-lycopenoic acid (lycopene metabolite) was observed in C57BI/6J mice which were induced with diethylnitrosamine following a high fat diet, supplemented with 10 mg/kg apo-10′-lycopenoic acid for 24 weeks. There was significant reduction in tumor multiplicity, volume and incidence. In terms of biomarkers, caspase-1, TNF-α, IL-6, NF-κB p65 protein expression, STAT3, Akt activation and cyclin D1 were suppressed significantly while an increment in SIRT1, deacetylation of SIRT1 and PARP cleavage were observed. In vivo experiments exhibited a similar outcome as shown in the in vitro test and thus, the author concluded that apo-10′-lycopenoic acid had the potential in inhibiting high fat diet induced hepatic tumorigenesis via suppression of inflammation and SIRT1 signaling activation [25].

One of the major key points from these experiments highlighted that the anti-cancer property of lycopene was often associated with inflammation suppression. Lycopene itself does possessed anti-inflammatory properties as discussed above and such property could actually help to create a synergistic effect towards its own anti-cancer activity. It is proposed that the downregulation of proinflammatory cytokines could aid in the resolution of inflammation, which reverses the formation of microenvironment suitable for tumorigenesis. Alleviation of inflammation could also greatly reduce the possibility of nearby cells being damaged by surrounding neutrophils or leukocytes during inflammation, minimizing the accumulation of DNA damage and thus, preventing the initiation of damaged induced cell proliferation and metaplasia.

## 6. Anti-Cancer Activities of Lycopene

### 6.1. Cell Culture Studies

All reviewed articles reported positive outcome in terms of the anti-cancer property of lycopene. The first published study on the topic of interest occurred in 2005, when SK-Hep-1 cells, a highly invasive hepatoma cell were treated with 1, 2.5, 5, 10, 20 µmol/L of lycopene. The result came out whereby lycopene was found to be able to suppress the cancerous cell migration and invasion, in a bell-shaped manner. Such suppression was negatively correlated with the expression of nm23-H1, a metastasis suppressor which was enhanced by lycopene. The author explained that the bell-shaped manner of lycopene could be due to the possibility of autooxidation of lycopene at high concentration and thus, searching for the correct concentration was crucial [16]. Two years later, by studying using the same cell line, treatment with 1–10 µM of lycopene significantly inhibited cell invasion, MMP-9, NF-κB, Sp1, IGF-1R and ROS production in cells. One important message from this study was that the antioxidant property of lycopene was only responsible for a minor role in its anti-cancer property as the anti-cancer property of lycopene was retained even after incubation with hydrogen peroxide [17]. A similar outcome was observed when the same cell line was treated with 0.1–5 µM of lycopene, induced with TGF-β for metastasis, whereby there was a reduction in NOX4 gene expression, NOX, ROS, MMP-9, MMP-2 and inhibition of cancerous cell migration, invasion and adhesion activity [19].

The anti-cancer property of lycopene was not restricted to only hepatoma cell, but also breast cancer cell. Highly aggressive breast cancer cell (H-Ras MCF10A, MDA-MB231) which had undergone treatment with lycopene exhibited inhibition of cell invasion, migration and proliferation, accompanied by a reduction in ERK and Akt, suggesting that both signaling pathways might play roles in the anti-cancer property of lycopene. In ER/PR+ MCF-7, HER2+ SK-BR-3 and MDA-MB-468 cell lines, treatment with lycopene for 168 h led to inhibition of cell cycle progression in G0/G1 stage, increased PARP cleavage, ERK 1/2, p21, Bax, reduced cyclin D1, phosphorylation of Akt, mTOR and no change for Bcl-xL [23]. The anti-cancer effect of lycopene on breast cancer was significant and thus, suggesting its potential in the prevention of breast cancer.

In human colon cancer cells (HT-29 cells), treatment with lycopene reduced cancerous cell invasion, expression of MMP-7, GSK-3β, ERK 1/2, AP-1, β-catenin, phosphorylation of Akt while increasing E-cadherin stabilization [21]. Lycopene was found to enhance the autophagy ability of gastric cancer cell line (HGC-27 cell lines) as observed by increment in the expression of LC3-1 accompanied by phosphorylation of ERK while decreasing tumor weight in Balb/c nude mice models carrying HGC-27 cells which were being fed with 20, 30 and 60 mg/kg lycopene per day [63].

In an investigation whether lycopene could play roles in DNA damage, it was revealed that incubation of lymphocytes from human blood with 10, 20 and 40 µM/mL lycopene before X-irradiation did cause significant decrement in DNA damage. However, such effect was only restricted to treatment with lycopene before induction of DNA damage via X-irradiation as lycopene treatment after irradiation failed to show such DNA protective effect. The author did emphasize that low doses are useful as compared to high doses of lycopene in this study [64]. The anti-cancer effect of lycopene was also observed in pancreatic cancer cells (PANC-1 cell line) whereby significant reduction of ROS, NF-κB and anti-apoptotic biomarkers (cIAP1, cIAP2 and survivin) was detected while an increment of caspase-3 and Bax:Bcl-2 ratio was noticed [24]. These evidences pointed out that via regulation of apoptosis, lycopene could reduce cell viability of cancerous cells and thereby, exhibiting its potential as a useful compound in reducing the incidence of pancreatic cancer.

In mouse epidermal cell line, JBG P+ (JB6 C1 41-5a), pretreatment with lycopene for 5 days, followed by incubation with TPA, with or without lycopene for 14 days significantly reduced colony formation and KEAP1 mRNA, while upregulating mRNA of SOD1, GSR, GPX1, CAT, GCLC, GCLM, NQO-1, HMOX1, nuclear NRF2 localization, LC3 and p62 protein levels. For in vivo part of the study, mice which were subjected to DMBA and TPA faced lower incidence rate, multiplicity of cutaneous papillomas, lower degree of increment in epidermal thickness, invasion of benign papillomas, 8-OHdG and 4HNE levels when lycopene was administered. The mice also exhibited higher survival rate, GSH/GSSG ratio, SOD, GR, GPx, CAT and mRNA of SOD1, GSR, GPX1, CAT, GCLC, GCLM, NQO-1 and HMOX1. The author concluded that lycopene was more effective as a pretreatment, especially during the promotion phase of induced tumors and NRF2 was required for the observed effect of lycopene-induced prevention against tumors. The activation of NRF2 signaling pathway might be related to the degradation of KEAP1 by p62 via autophagy-lysosomal pathway [65].

There were two metabolites of lycopene which were under study for their anti-cancer effect, which include apo-10′-lycopenoic acid and apo-8′-lycopenal. Treatment of NHBE cells (human bronchial epithelial cells, BEAS-2B-immortalized normal bronchial epithelial cells and A549 (non-small cell lung cancer cells) with apo-10′-lycopenoic acid managed to increase p21, p27 protein levels and reduce cyclin E level which resulted in inhibition of cell cycle progression from G(1) phase to S phase. Apo-10′-lycopenoic acid also caused a significant increment of RAR-beta which is involved in the binding of retinoic acid (a biological active form of vitamin A). The evidence of anti-cancer activity of apo-10′-lycopenoic acid was strengthened when there was a significant reduction of tumor multiplicity observed in the A/J mouse model injected with NNK (induction) and supplemented with 10, 40, 120 mg/kg of apo-10′-lycopenoic acid [14]. In another independent study investigating the mechanism behind such anti-cancer activity, the result pointed out that apo-10′-lycopenoic acid may increase NRF2, HO-1, NAD(P)H dehydrogenase (quinone 1), GSTs, GCL and GSH levels in BEAS-2B cells, suggesting that lycopene may exhibit both anti-carcinogenic and antioxidant activity via NRF2 activation and induction of detoxifying enzymes [66]. The anti-cancer activity of apo-10′-lycopenoic acid was observed in human liver THLE-2 and HuH7 cells whereby increment in SIRT1 enzyme level, p21 and apoptosis activity accompanied by a reduction in cyclin D1 protein. Under the same study, C57BI/6J mice which were induced with diethylnitrosamine following a high fat diet, supplemented with 10 mg/kg apo-10′-lycopenoic acid for 24 weeks showed significant reduction in tumor multiplicity, volume and incidence. In terms of biomarkers, caspase-1, TNF-α, IL-6, NF-κB p65 protein expression, STAT3, Akt activation and cyclin D1 were suppressed significantly while an increment in SIRT1, deacetylation of SIRT1 and PARP cleavage were observed. In vivo experiment exhibited similar outcome as shown in the in vitro test and thus, the author concluded that apo-10′-lycopenoic acid had the potential in inhibiting high fat diet induced hepatic tumorigenesis via suppression of inflammation and SIRT1 signaling activation [25].

In a study investigating both lycopene and apo-8′-lycopenal (lycopene metabolite) using SK-Hep-1 cell line, treatment with 1, 2.5, 5, 10 µM apo-8′-lycopenal and 10 µM of lycopene significantly inhibited the invasion and migration of cancerous cell. Apo-8′-lycopenal reduced the expression of MMP-2, MMP-9, Rho GTPase via inhibition of ERK/p38 and PI3K-Akt pathway while increased expression of metastasis suppressor nm23-H1, TIMP-1, TIMP-2. One interesting point was mentioned by the author whereby the anti-cancer activity of apo-8′-lycopenal was higher than lycopene, suggesting that this metabolite could play an essential role in the observed anti-cancer property exhibited by lycopene [18]. In human HepG2 cells, treatment with 1, 5, 10 µM of apo-8′-lycopenal resulted in inhibition of cancerous cell invasion, migration and changes in biomarkers (increment in NRF2 accumulation, HO-1 and NQO-1, decrement in KEAP1). The author discovered that ERK/p38-NRF2 pathway may be involved in activation of phase II detoxifying enzyme expression (HO-1, NQO-1) and the time taken for NRF2 accumulation for lycopene was longer than apo-8′-lycopenal, indicating that this metabolite might partially be involved in chemopreventive effects of lycopene due to its relatively short NRF2 accumulation time [15] (Table 1).

### 6.2. Animal Studies

Most of the reviewed articles reported a positive the outcome on anti-cancer activity of lycopene while only two studies reported that lycopene had no significant chemopreventive effect. The first study of anti-cancer activity of lycopene on animals took place as early in 1995, whereby high mammary tumor strain of SHN virgin mice were used. In this study, the mice were fed with either control diet or diet containing 5 × 10^−5^% of lycopene. It was discovered that mice fed with lycopene had suppressed mammary tumor development and reduced level of TYMS, serum FFA and prolactin [67]. Similar outcome was also reported in a study using rat mammary tumor model but the major difference was the application of lycopene-enriched tomato oleoresin (LETO) instead of pure lycopene. First, rats were injected with 10 mg/kg of LETO twice per week for two weeks, followed by induction with 7, 12-dimethyl-benz[a]anthracene (DMBA) and the injection of 10 mg/kg of LETO lasted for a total of 16 weeks. The administration of LETO managed to increase both plasma and hepatic lycopene and thus, rats which received such supplementation developed less and smaller tumor, proving the effectiveness of lycopene in protecting against mammary cancer [68].

Lycopene supplementation (1, 20 mg/kg BW; 2 times per week for 12 weeks) in nude mice injected with SK-Hep-1 cells managed to significantly reduce MMP-2, VEGF, PCNA, MMP-9, suppress tumor metastasis, mean number of tumors, tumor cross-sectional area while increase nm23-H1. A dose-dependent relationship between lycopene and the observed effect was reported [22]. In Lewis lung carcinoma cells, the in vitro experiment on treatment with 10, 20 or 40 µM of lycopene caused increased IFNβ, IFNγ, IRF1, IRF7, CXCL9, CXCL10, pJAK and pSTAT3 mRNA expression while suppressed mRNA expression of DMNT3a, methylation levels of promoters (IRF1, IRF7) and PD-1 as induced by IFNγ via suppression in phosphorylation of Akt. There was no observable change in gene expression of DNMT1 and DNMT3b. Under the same study, an experiment on C57BL/6 mice with lycopene (40 mg/kg) administered intraperitoneally for 3 days resulted in the reduction of tumor volume, weight, IL-4, IL-10, gene expression of DMNT3a and methylation levels of promoters (IRF1, IRF7). There was a significant increment observed in IL-2, IFNγ, CD4^+^:CD8^+^ T cells ratio, percentage of IFNγ^+^/CD8^+^ T cells, percentage of perforin^+^/CD8^+^ T cell, percentage of granzyme B^+^/CD8^+^ T cell, gene expression of IFNβ, IFNγ, IRF1, IRF7, CXCL9 and CXCL10. Similar to the in vitro study, no change was observed in gene expression of DNMT1, DNMT3b, IRF3 and IRF8 [69].

The anti-cancer effect of lycopene was also observed in liver whereby significant reduction of GGT^+^, GST^+^ foci size and liver volume fraction occupied by foci were noticed in male weanling rats which had undergone induction using diethylnitrosamine (DEN) or 2-nitropropane (2-NP), followed by lycopene (300 mg/kg) administered intraperitoneally for 3 to 4 weeks. No change in the number or size of preneoplastic liver foci was detected and the author found out that such activity was unrelated to the antioxidant property of lycopene, but rather its effect in modulating CYP2E1 [70]. In resistant hepatocyte model of hepatocarcinogenesis Wistar rats, 70 mg/kg BW of lycopene increased liver carotenoid concentration and reduced number, size and area of GST^+^ preneoplastic lesions and hepatic DNA strand breakage. However, no effect was seen on incidence, total number and multiplicity of hepatocyte nodules [71]. In another similar study, lycopene was fed together with high fat diet after injection with DEN and lycopene was able to reduce the number of GST^+^ hepatic foci and PCNA, cyclin D1 levels, via inhibition of ERK pathways and NF-κB transcription. A significant increment was observed for HO-1 and NRF2 level whereby no change was detected for TNF-α, IL-1β, IL-12 and CYP2E1. Surprisingly, high fat diet feeding with tomato extract caused reduction in proinflammatory cytokines TNF-α, IL-1β, IL-12 and CYP2E1 enzyme, which were unaffected by lycopene. Thus, the author concluded that a combination of both tomato extract and lycopene was able to prevent hepatocarcinogenesis via a different mechanism [72]. In BCO2-knockout male mice, lycopene supplementation for 24 weeks in combination with high fat diet resulted in an increment in hepatic lycopene, miR-199a/b, miR214 and decrement in hepatocellular carcinoma incidence, multiplicity, endoplasmic reticulum stress-mediated unfolded protein response, ER(UPR) and oncogenic biomarkers such as Met mRNA, β-catenin and mTOR1. With the same experimental design but in wild type mice, lycopene significantly exhibited anti-cancer property, mainly by inhibition of proinflammatory signaling as seen in a significant reduction in phosphorylation of NF-κB p65, STAT3, IL-6 and suppressed inflammatory foci. The difference in terms of the observed anti-cancer outcome suggested that BCO2 expression may cause a difference in terms of the pathway taken to exhibit anti-cancer property [62].

In gastric cancer, male Wistar rats induced with N-methyl-N′-nitrosoguanidine (MNNG) + saturated NaCl, followed by lycopene administration resulted in reduced gastric carcinomas accompanied by increase in GSH and antioxidant enzymes GPx, GST and GR. The author proposed that the ability of lycopene in modulating antioxidant enzymes might be the major contributor to its anti-cancer activity [73]. In another independent experiment using the same animal model and similar experimental design, lycopene managed to reduce tumor burden, Bcl-2 levels and increase caspase-8, liver GSH, stomach GPx and GSH and GPx activities in liver and erythrocytes. No change was detected in stomach and erythrocytes GSH, liver and erythrocytes GPx, GPx activities in stomach, Bax and Bim levels [74]. The new found capability of lycopene in the modulation of apoptosis-associated protein, aside from its antioxidant property suggested that the mechanism behind its anti-cancer activity could be a combination of simultaneous activation of various pathways [26]. Recent discoveries pointed out that modulation of the immune system via cytokines and antibodies might play roles in the anti-cancer property of lycopene. Such claim arose from a study focusing on MNNG gastric cancer rat model whereby administration of 50, 100 or 150 mg/kg body weight of lycopene caused an increment in antioxidant enzymes as expected (SOD, CAT, GSH-Px) and increment in cytokines (IL-2, IL-4, IL-10, TNF-α) and antibodies (IgG, IgA, IgM). Noticeable reduction in MDA and IL-6 were detected under the same study. The author suggested that the upregulation of both antioxidant status and the immune system might play roles in the anti-cancer activity of lycopene [61].

In terms of ovarian cancer, in vitro study using OV-MZ-6 cells with lycopene treatment (2, 5 µM) caused a significant reduction in ITGA5 and pERK 1/2 and no change in ITGB1, total ERK and vimentin. Under the same study, there was an in vivo experiment using ovarian cancer-bearing mice which were separated to lycopene prevention and lycopene treatment group. In the prevention group, lycopene (0.75 mg/mL) was fed 2 weeks before implantation of cell-seeded hydrogels while in the treatment group, lycopene was fed 4 weeks after the implantation surgery. In the lycopene prevention group, lycopene significantly reduced metastatic load, Ki67, ITGA5B1, ITGA5, ILK, ITGB1, FAK, MMP-9. Reduction in serum and ascites CA125 and EMT markers in metastatic tissue were reported. The reduction in MMP-9 was restricted to only metastatic tissue but not tumor tissue. Lycopene had no effect upon tumor load, serum and ascites MMP-9. The in vitro study pointed out that the reduction of ITGA5 could be related to reduced pERK activity, suggesting that reduced activation of MAPK was the key to all these observed effects. In the lycopene treatment group, there was reduction in tumor load, Ki67, ITGA5 ITGA5B1, ascites CA125 but there was an increment in MMP-9. No significant change was reported for ITGA5, ILK, ITGB1, FAK, serum and ascites MMP-9 and serum CA125 [75]. Through this experiment, lycopene, when being consumed in a preventive manner was found to be effective towards metastatic cancerous tissue but not towards the primary tumor. On the other hand, even though lycopene treatment managed to reduce tumor load, its effect was relatively weaker to what had been observed in the prevention group. In laying hens, 200, 400 mg/kg per kg diet of lycopene was able to reduce incidence, number and size of ovarian tumor. The rate of adenocarcinoma and MDA level were lowered and these effects could be a result of the inhibition of NF-κB transcription and expression of STAT3. A noticeable increment was observed for NFE2 and HO-1. In this experiment, the antioxidant property of lycopene could contribute to its anti-cancer activity due to noticeable changes in oxidative stress markers [76].

In Sprague Dawley rats, induction using N-methylnitrosourea intrarectal for a week, followed by a week of lycopene administration resulted in reduced aberrant crypt foci development, suggesting the potential of lycopene in the prevention of colon carcinogenesis [77]. It was interesting to point out that often times, food source which was rich in lycopene such as tomato was more useful in the exhibition of anti-cancer activity. According to research done on F344/NSlc rats, feeding of diluted tomato juice with 17 ppm lycopene caused a significant reduction in colon cancer incidence while feeding of 17 ppm pure lycopene failed to exhibit such a result. In this study, the concentration of lycopene in tomato juice did make a difference as feeding with tomato juice with only 3.4 ppm resulted in non-detectable lycopene amount in colon mucosa, unlike the group fed with 17 ppm lycopene in diluted tomato juice, which had detectable amount of lycopene (0.02 µg/g) found in colon mucosa [78]. In a study using colon carcinogenesis Sprague Dawley rat model, lycopene treatment after induction by azoxymethane caused suppression of aberrant crypt foci, preneoplastic lesion and biomarkers such as COX-2 and iNOS expression [60]. Research on CD-1 mice in AOM-DSS model showed that lycopene administration (20, 50 mg/kg) could reduce inflammation incidence and positive rates of IGF-2, IGFBP3 at low dosage and IGFBP2 at high dosage. It could also increase lymphocyte infiltration but it caused variability in growth factor according to dosage. One noticeable variability was that at low dosage, even though there was a decrement in positive rates of IGF-2 and IGFBP3, a significant increment of positive rates was detected for IGF-1R, IGF2BP1 and IGFBP2. Similarly, for high dosage, there was a significant spike in positive rates of IGF-1, IGF-2 and IGFBP3 despite a significant reduction in IGFBP2. There was no change in the number of tumors or adenocarcinomas incidence but there was noticeable focal necrosis present in colonic tissue [79]. The anti-cancer effect of lycopene in this experiment could be said to be moderate as there were both positive and negative outcomes, accompanied by its ineffectiveness in reducing the number of tumors and adenocarcinomas incidence. One main point from this experiment was that lycopene could apparently help to suppress inflammation but its effect on colon tumorigenesis was still debatable.

Interestingly, in a multiorgan carcinogenesis B6C3F1 mice model, lycopene (25/50 ppm) administration for 21 weeks after combinational induction with diethylnitrosamine (DEN), N-methyl-N-nitrosourea (MNU) and 1,2-dimethylhydrazine (DMH) led to reduction in incidences and multiplicities of lung adenomas and carcinomas but the effect was restricted to male mice fed with 50 ppm lycopene and water. However, no effect was seen for female or aberrant crypt loci and tumors in both colon and kidney among groups [80]. This experiment pointed out that the chemopreventive effect of lycopene in multiorgan carcinogenesis model was limited and only effective for male, specifically for lung carcinogenesis.

There were two studies which reported the ineffectiveness of lycopene in exhibiting anti-cancer effect and one of such experiment occurred in 2001. In this experiment, hepatocellular carcinoma LEC rats were the subject under study and they were given diet containing 0.005% of lycopene from 6 weeks of age to 76 weeks age. The reported outcome of the study was that lycopene failed to cause any significant change in number, mean area, percentage area of GST-P-+ focal lesions in liver, Alpha-fetoprotein (AFP) and cumulative survival rates of rats. However, a depletion of iron concentration was noticed in liver. The author hence concluded that long term administration of lycopene had no effect in reducing risk of hepatocarcinogenesis in LEC rats [81]. Another research placed the focus on the difference between the chemopreventive effect of both pure lycopene and lycopene-rich tomato carotenoid oleoresin (TCO) in rat mammary tumor model. Rats were first supplemented with 250, 500 ppm lycopene or TCO, followed by initiation with N-methylnitrosourea (NMU) for 7 days, and the experiment lasted for 18 weeks. As a result, no significant change in tumor incidence, latency, multiplicity, volume or total tumors per group was reported for all groups. The author supported the fact that lycopene was ineffective in preventing breast cancer as reported by some epidemiological reports [82]. One point from this experiment worth mentioning was that supplementation with TCO managed to increase serum lycopene concentration in a higher manner than supplementation with pure lycopene under the same lycopene concentration (Table 2).

### 6.3. Clinical Trials

A total of 15 articles involving the clinical trials on the anti-cancer effect of lycopene were reviewed and it was reported that 11 out of these 15 studies reported positive outcomes and four of them reported that lycopene had no effect upon reducing the risk of cancer. The first related cohort study came in 1995 whereby 47,894 human subjects who were initially free of diagnosed cancer were recruited in 1986 and given validated semiquantitative food-frequency questionnaire as a mean of dietary assessment. Follow-up questionnaires were given in 1988, 1990 and 1992 and the data collected were analyzed. Data analysis pointed out that only lycopene was found to be able to reduce the risk of prostate cancer. Four food items which were known for their high lycopene content such as tomatoes, tomato juices, tomato sauces and pizza were also able to reduce risk of prostate cancer. The combined intake of these four items managed to establish an inverse association with risk of prostate cancer [83]. The author then suggested that lycopene or tomato-based food might be beneficial for prostate cancer and this became the first evidence of anti-cancer property of lycopene among humans. A similar outcome had been reported in another cohort study comprised of 47,365 participants who were given dietary questionnaire for dietary assessment in 1986, 1990 and 1994. The pooled analysis showed that lycopene was able to reduce the risk of prostate cancer but the association was considered moderate after controlling for fruits and vegetable consumption and olive oil consumption (marker for Mediterranean diet) [84]. In terms of plasma lycopene, a case–control study nested within a prospective Health Professionals Follow-up Study with 450 incident prostate cancer cases reported that higher plasma lycopene could reduce risk of prostate cancer but such association was only restricted to older participants without family history [85].

In a clinical trial, 26 male patients with prostate cancer with 14 being in stage T1 and 12 being in stage T2 were recruited to participate in a trial examining the potential of lycopene as a chemopreventive agent. Fifteen mg of lycopene supplementation managed to reduce plasma prostate-specific antigen (PSA) and increase connexin 43, a component of gap junction which allows intercellular communication between cells. There was no significant change in Bcl-2 or Bax. The author mentioned that lycopene supplementation could possibly help in controlling the growth of prostate cancer. However, the small sample size was also the major limitation of this study, causing the reliability of the result to be questionable [86]. Another randomized placebo-controlled study shared a similar outcome whereby 30 mg of lycopene per day among 32 patients with localized prostate adenocarcinoma significantly reduced serum PSA and leukocyte 8OHdG while increased both serum, prostate lycopene concentration and apoptotic index of hyperplastic and neoplastic cells. These evidences pointed out that lycopene could reduce DNA damage occurred in both leukocyte and prostate tissue but whether such reduction could be beneficial or detrimental for cancer cells still requires further research [87]. Decrement of PSA was also observed in a nutritional intervention among 79 prostate cancer patients but instead of pure lycopene, lycopene rich tomatoes were used for the study. Subjects were fed with tomato products containing 30 mg of lycopene throughout the whole study and a significant reduction in PSA level was detected [88].

Meta-analysis of 11 cohort studies and 6 nested case–control studies pointed out that high tomato intake was negatively correlated with the incidence of prostate cancer and lycopene had a modest effect in the prevention of prostate cancer [89]. The result from this meta-analysis was supported by another meta-analysis involving 26 studies with 17,517 cases of prostate cancer from 563,299 participants whereby lycopene (9–21 mg/day) or plasma lycopene (2.17–85 µg/dL) was able to reduce the risk of prostate cancer [90].

Apart from prostate cancer, lycopene seemed to be able to help with breast cancer, as reported in a pooled analysis of 18 prospective cohort studies in 2012 using the interval collapsing method. In this study, lycopene was found to have a protective effect towards ER^-^/PR^+^ or ER^−^/PR^−^ breast cancer [91]. In a study involving 521 an women with breast cancer, analysis of plasma using HPLC showed that there was inverse association between serum lycopene and risk of breast cancer among premenopausal women and all ER/PR subtypes [92]. In lung cancer, there was a nonlinear dose-dependent association between lung cancer incidence and plasma lycopene as reported in a meta-analysis analyzing 17 prospective studies with 3603 cases involving 458,434 participants [93]. The inverse association mentioned was even stronger at low plasma lycopene concentration (20 µg/100 mL) and a weaker association above this concentration.

The first study to report non-significance results occurred in 1990. It was a nested case–control study whereby serum was obtained from 25,802 persons in 1974 were used. Serum of 103 men who developed prostate cancer during the subsequent 13 years was compared with the serum of 103 control subjects matched for age and race. There was no significant association between serum lycopene and risk of prostate cancer [94]. A similar result was obtained in the study involving 209 prostate cancer cases with 228 control, both black and white men in the US aged between 40 and 79 years old. Analysis of serum carotenoids revealed that lycopene had inverse association with prostate cancer which failed to reach statistical significance. Lycopene only showed significant inverse association particularly for aggressive disease [95]. In terms of lycopene consumption, a cohort study with 6.3 years of follow-up involving 58,279 men aged 55–69 years old (642 prostate cancer cases) showed that lycopene consumption had no effect upon the risk of prostate cancer via dietary assessment using semi-quantitative food-frequency questionnaire [96]. A 3-month randomized, double blinded clinical trial involving 69 men with a a favourable risk for prostate cancer who took 30 mg of lycopene per day yielded no change in IGF-1 and COX-2 levels in their prostate tissue gene expression [97]. The main interest of this experiment was not focusing on the chemopreventive effect of lycopene, but rather the effect of lycopene in modulating biomarkers related to cancer and inflammation. Thus, the outcome of this study was insufficient to justify the fact that lycopene was not effective in exhibiting anti-cancer property as pathway taken to suppress cancer cell was not only restricted to the parameters under study (Table 3).

Results from cohort studies had been conflicting whereby reports from studies with large sample sizes inclined towards a direction whereby lycopene was not able to reduce risk of cancer or lycopene could only have moderate effect on cancer risk reduction [83,84,94,95,96]. The outcome from meta-analysis of cohort and case–control studies was positive whereby it was reported that lycopene could reduce risk of prostate cancer, lung cancer and breast cancer, especially at low plasma lycopene concentration [89,90,91,93]. High level of evidence from randomized controlled trials suggested that lycopene could be beneficial for cancer as seen in increment in apoptotic index among hyperplastic and neoplastic cells and suppression of PSA in prostate cancer patients [86,87,88]. However, in randomized controlled trials, lycopene failed to cause any significant change towards Bax protein and IGF-1, as opposed to what had been shown in cell culture and animal studies [86,97]. Such limited evidence from randomized controlled trials could not help us to deduce whether lycopene was effective in exhibiting anti-cancer activity among human.

## 7. Immunomodulatory Effects of Lycopene

The earliest evidence came in 2004 when lycopene was able to modulate dendritic cell response by downregulation of CD80, CD86 and MHC II molecules expression, which are the common protein found on surface of dendritic cells. In vivo experiment further revealed that the effect of lycopene could be extended to decreased stimulation of T cells, accompanied by reduced expression of IL-2 and IL-12, the key stimulators of T cells. It was suggested that such effect was a result of MAPK/ERK signaling pathway inhibition (ERK1/2, p38, JNK) and reduced transcription of NF-κB. These evidences gave a direction whereby lycopene could suppress the maturation of murine dendritic cells and cell-mediated response under stimulation of LPS [98]. Mast cells are a type of granulocytes commonly known to be involved in allergic reaction and anaphylaxis. It plays a major role in inflammation as mast cell degranulation could release mediators or compounds which trigger an inflammatory response. Lycopene pretreatment with basophilic leukemia cell line suppressed mast cell degranulation but such activity was most probably not a direct result of lycopene cellular uptake as there was no correlation found between cellular carotenoids content and anti-degranulation activity. This suggests that the effect of lycopene in immune system modulation is not as simple as absorption and execution and it could be a result from a complicated network consisted of simultaneous activation of various immunomodulatory pathways [99]. In barrow and gilt finishing pigs, 0, 12.5, 25, 37.5, 50 mg/kg of lycopene administration followed by immunization using 1 mg BSA caused increased lymphocyte concentration and anti-BSA IgG, reduced neutrophil concentration and eosinophil while causing no change in basophil and monocyte [100]. Changes in lymphocyte concentration could be an indicator of activation of cell-mediated or humoral immune response and thus, lycopene was proposed to be able to influence immune response and the production of antigen-specific antibodies.

One study provided a lead whereby lycopene could activate the immune system, especially adaptive immunity in the process of eradicating cancerous cells. In this experiment, lycopene managed to enhance IFNβ, IFNγ, IRF1, IRF7, CXCL9, CXCL10 while suppressing IL-4, IL-10, DMNT3a, methylation of IRF1, IRF7 promoters and most importantly, the tumor volume. Increment of CD4^+^/CD8^+^ T cells ratio, percentage of IFNγ^+^/CD8^+^ T cell, percentage of perforin^+^/CD8^+^ T cell and percentage of granzyme B^+^/CD8^+^ T cell were reported, accompanied by no significant change in DNMT1 and DNMT3b [69]. Compiling these evidences depicted that lycopene could enhance activation and differentiation of T cells and T helper cells Th1/Th2 drift via suppression of IL-4 and upregulation of IFNγ, most probably by its ability to modulate cytokines, chemokines and interferons. Interferons had been known to be a central regulator for anti-tumor immunity whereby it had both anti-tumor and pro-tumor activity. In anti-tumor activity, interferons can increase antigenicity of tumor cells by upregulation of MH class I molecules and increase cytotoxic activity of both NK cells and cytotoxic T cells [101]. Suppression of DNMT3A could suppress DNA methylation of IRF1 and IRF7 promoters and thereby permitting transcription [102]. IRF1 was reported to be antioncogenic due to its ability to mediate apoptosis via induction of caspase-1, upregulation of caspase-8 and suppression of CDKs and survivin [103] while IRF7 was able to increase NK cell cytotoxic activity on cancerous cells via upregulation of IFNβ and inhibit bone metastasis [104].

## 8. Effects of Lycopene on Oxidative Stress and Liver Enzymes

Lycopene was long known for its antioxidative property in fighting free radicals and it was able to suppress the production of highly reactive free radicals such as ONOO^−^ [74] and reactive oxygen species (ROS). The antioxidant activity of lycopene on the reduction of ROS was highly dependent on the concentration of administered lycopene [105]. Such property was often accompanied by anti-inflammatory activity and whether the anti-inflammatory activity of lycopene was an indirect result of its antioxidant activity or lycopene does possess both anti-inflammatory and antioxidant property still requires further research.

In terms of oxidative stress, lycopene significantly reduced generation of ROS, increased GSH/GSSG ratio, expression of NOX4, NOX, KEAP1 while increased expression of NRF2, HO-1 and detoxifying enzymes such as NAD(P)H dehydrogenase (quinone 1)/NQO1, GSTs, GCL, GPx, GR and GSH [15,17,19,24,26,53,54,61,65,66,72,73]. By combining these evidences, it was apparent that lycopene could affect NRF2/ARE signaling pathway. NRF2/ARE signaling pathway is the major regulator of phase II detoxifying enzymes and antioxidant genes which helps to protect cells from inflammation and oxidative stress. Suppression of KEAP1, which is a known inhibitor of NRF2 transcriptional activity via ubiquitination and proteosomal degradation, could enhance NRF2 transcriptional activity in KEAP1 dependent NRF2 regulation. The suppression of KEAP1 could be related to p62 as p62 which is commonly being known as autophagy substrate and used as a reporter of autophagy activity competes with NRF2 for KEAP1 binding. Such competition eventually free NRF2 to be accumulated in nucleus for transcription [106]. Successful transcription of NRF2 could increase gene expression of antioxidant response element (ARE), which includes, HO-1, GST and NQO1 [107]. The uprising of AREs can further enhance the antioxidant activity of cells and anti-inflammatory activity as well, by decreasing the proinflammatory cytokine expression via inhibition of NF-κB pathway. The antioxidative activity in cancer could also be observed in upregulation of both SOD, CAT and suppressed MDA production under lycopene administration to gastric cancer rat model [61,108].

Apart from antioxidant activity, the capability of lycopene in modulating the liver enzymes was worth mentioning. Researches pointed out that lycopene consumption could suppress expression of ALT, AST, LDH, gamma-glutamyltransferase (GGT) and production of malondialdehyde (MDA) and 4HNE but it had no effect on acute phase protein, CRP [65,109,110,111,112]. The effect of lycopene on MDA had been inconsistent as dosage of lycopene administered and the subject of study varied among studies [109,110,112,113]. High concentration of lycopene (3.3 mg/kg body weight) feeding with alcohol to rats could increase hepatic cytochrome P4502E1 (CYP2E1) levels but it could also increase severity of inflammatory foci simultaneously. Such change was not observable in group fed with low concentration of lycopene. The author later warned about the safety of practice involving alcohol consumption and high concentration lycopene supplementation [114]. Modulation of CYP-4502E1 was observed in anti-cancer study as well whereby such modulation was the main contributor of the anti-cancer property possessed by lycopene, but not the antioxidant activity of lycopene [70].

## 9. Selective Anticancer Activities of Lycopene

### 9.1. Carotenoids of the Same Kind with Different Fate (Lycopene and Beta-Carotene)

Both lycopene and beta-carotene are classified under the same carotenoid family. Lycopene is an aliphatic hydrocarbon with molecular formula C_40_H_56_. It is dark red in color with waxy consistency found commonly in tomatoes. It is an open chain polyene with 13 double bonds which has the longest chain length as compared to other carotenoids due to the linear array arrangement of its 11 conjugated carbon-carbon double bonds. Unlike beta carotene, it does not possess any vitamin A activity due to its symmetrical planarity [28]. Beta-carotene is also a member of carotenoid family which is often seen as a red and orange colored pigment. It shares the same chemical formula with lycopene and it is the major carotenoids in human diet. It is a significant source of vitamin A [115] with the highest provitamin A activity and its deficiency is highly similar to that of vitamin A deficiency, such as blindness and xerophthalmia [116].

In comparison, both lycopene and beta-carotene are antioxidants, with lycopene being the best of carotenoids in an in vitro experiment setting. However, in vitro experiment setting showed that beta-carotene could be prooxidative, given at a high concentration and partial pressure but there was no reported evidence of lycopene being prooxidative. Thus, beta-carotene was found to be able to contribute to smoking oxidation products while there was no reported data for lycopene. Lycopene does not possess provitamin A activity and therefore, there was no evidence of the conversion of lycopene to retinoids, unlike beta-carotene. One interesting point worth mentioning is that beta carotene was often involved with a bad reputation of increasing the risk of lung cancer [117]

Beta-carotene first gained its bad reputation in lung cancer when several large intervention trials (ATBC, CARET) reported that beta-carotene supplementation was correlated with adverse effects in smokers. In heavy smokers, beta-carotene supplementation seemed to increase lung cancer incidences among them while having no protective effects towards nonsmokers and former smokers [118,119]. Two large prospective cohort studies focusing on epidemiological data reached similar consensus whereby beta-carotene was not associated with lung cancer risk in never smokers [120] while beta-carotene showed dual association within subjects whereby beta-carotene actually increased risk of lung cancer in smokers [121]. One research pointed out that although there was inverse risk association of lung cancer for beta-carotene in nonsmokers, it was statistically insignificant.

A few hypotheses were proposed for the situation observed in smokers. Oxidative stress was one of them whereby it was speculated that beta-carotene whenever present in high concentration in lungs may have the tendency to have prooxidative activity, which eventually leads to oxidative DNA damage. This could set off the co-carcinogenic action of beta-carotene via epigenetic mechanism which were discovered in rats whereby high doses of beta-carotene induced several cytochrome p450 (CYP) enzymes. Beta-carotene is a well-known precursor of retinoic acid, which is the main component behind retinoic acid signaling pathways which governs cell proliferation and differentiation. Another hypothesis came in whereby it was proposed that in places with high oxygen concentration such as lungs, high concentration of beta carotene could lead to oxidative cleavage or degradation products such as apocarotenals. This could cause disruption towards retinoic acid signaling pathways which sets off the carcinogenesis process [122].

The same situation surprisingly could not be applied to lycopene. For lycopene, increased intake of lycopene led to a significant reduction of cancer risk, even in smokers [123]. Another study revealed one interesting point whereby smoking failed to alter the concentration of lycopene but most of the other carotenoids’ concentrations were affected [124]. Thus, it was deduced that the anti-cancer of lycopene would be more robust when it comes to lung cancer prevention and may play a special role in lung cancer prevention [125].

### 9.2. Lycopene Is Selective against Lung Adenomas and Carcinomas

The ability of lycopene in terms of anti-cancer activity could be selective as observed in an experiment using a multiorgan carcinogenesis mice model. In this model, only male mice administered with 50 ppm of lycopene had lower incidence and multiplicities of lung adenomas and carcinomas while having no significant change found in tumors present in colon and kidney. Such evidence suggested that the anti-cancer activity behind lycopene could be highly effective and selective towards tumor cells in lungs, rather than any other part of the bodies but such a hypothesis required more research and scientific evidence. As compared to beta-carotene, which is classified under the same carotenoids family as lycopene, lycopene seemed to be able to suppress cancerous cells in lungs instead of increasing the incidence of lung cancer like how beta-carotene did [118,121,126]. Observed effects on males could indirectly provide a direction whereby lycopene may modulate testosterone levels, or more specifically, suppress serum testosterone [127].With regard to the reason behind the selective ability of lycopene towards lungs, it could be related to the role of retinoic acid in lungs and the types of epithelial cells present. Retinoic acid had long been known to be beneficial for epithelial cells as it is essential for epithelial cell differentiation. Severe deficiency in vitamin A was related to the formation of squamous metaplastic lesions [128] and retinoic acid was found to play a major role in lung development as retinoic acid signaling influences lung specification, branching, morphogenesis and alveolarization. Developing lungs are highly sensitive to retinoic acid levels as retinoid acid signaling could affect mesenchymal gene expression [129]. This could be related to the selective ability of both lycopene and beta-carotene towards lungs as retinoic acid plays major role in lungs development and slight change in retinoic acid could impact lungs greatly. In terms of epithelial cells, the distinct epithelial cells found in alveoli, specifically squamous epithelial cells which become the barrier for gaseous exchange, had greater potential for proliferation and thus, higher possibility for being cancerous as one of the most common cell origins of lung cancers are squamous cell carcinomas [130]. Combining these information led to a hypothesis whereby the selective ability of lycopene towards lung cells is a combinational result of the high possibility of carcinogenesis of squamous cells in lungs and the high sensitivity of these cells towards retinoic acid change.

One of the metabolites of lycopene, acycloretinoic acid is actually an analog of retinoic acid and it was found to be able to activate retinoic acid receptor (RAR). However, it was considered as a weak activator of RAR as the concentration required for activation needs to be much higher than that of all-trans retinoic acid, ATRA (the common activator of RAR) [131,132]. Researchers found that testosterone may have an effect on RAR signaling in prostate carcinoma cells whereby testosterone could increase RAR alpha and gamma expression, which could be attenuated by retinoic acid [133]. The complex interaction behind lycopene, its metabolites, RAR and testosterone may be the reason be related to selective ability of lycopene towards prostate cancer. The attenuation of RAR expression by retinoic acid was considered abnormal as retinoic acid was known for its anti-cancer activity via activation of RAR or RXR [134]. Though RAR had been known for its tumor suppressive ability, such a statement could not be applied to all cancerous cell lines as high expression of RAR was correlated with the progression of breast cancer and colorectal cancer [135,136]. Such an observation could be related to the difference in terms of the involvement of different signaling pathways behind the pathogenesis of various cancer. One of the hypotheses proposed would be RAR expression may be beneficial for some cancerous cells and detrimental at the same time, depending on the nature of pathogenesis of cancer as RAR and RXR were the main receptors behind RA response elements (RARE), which is essential for cell growth and development, regardless of whether the cell is normal or cancerous [137]. The difference observed between lycopene and beta-carotene could be related to the nature of lycopene metabolite, acycloretinoic acid being a weak activator of RAR as compared to ATRA (metabolite of beta-carotene). In hypothesis, given the same concentration of both metabolites, ATRA would be more likely to cause high expression of RAR, which could be beneficial for certain cancerous cells. In contrast, acycloretinoic acid would be less likely to behave as such, leading to an average expression of RAR which could neither be beneficial nor detrimental to cancerous cells.

**Figure 2:**
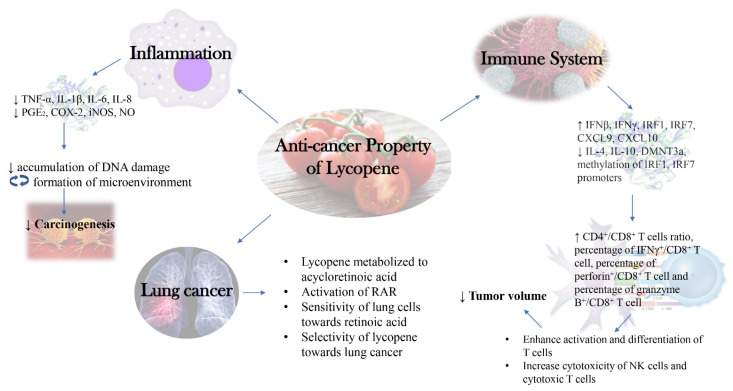
New Insights into Molecular Mechanism behind Anti-cancer Activity of Lycopene.

A summarized diagram depicting the new insights into the molecular mechanism behind anti-cancer activity of lycopene as discussed in this review was presented below (Figure 2).

## 10. Conclusions

It was a well-known fact that lycopene did possess anti-cancer activity from previous research whereby consumption of lycopene could benefit humankind, especially for those suffering from prostate cancer. Previous literature highlighted that lycopene is useful in suppressing cancerous cell proliferation, migration, invasion and enhancing apoptosis via modulation of cancer-related genes such as MMP and Bcl2 family. Cell cycle arrest is another target of lycopene whereby lycopene resulted in a halt in cell cycle progression from G_0_ to G_1_ phase and cell cycle progression from G_1_ to S phase. In this review, we discussed the new perspectives and insights of the molecular mechanism of how lycopene could exhibit its anti-cancer activity. The first new insight discussed builds a bridge between inflammation and cancer whereby it was deduced that part of the anti-cancer activity observed in lycopene could be related to its anti-inflammatory activity. As inflammation is well controlled and resolved, it greatly minimizes the possibilities of DNA damage caused by immune cells which are commonly observed in chronic inflammation. The second insight linked the ability of lycopene to modulate the immune system with its anti-cancer activity. It was proposed that lycopene could have the ability to activate our immune system to target cancerous cells and obliterate them before they can threaten our health, as seen in the activation of cytotoxic T cells. This review also did look into both lycopene and beta-carotene and their different effects in lung cancer risk association. Though they are from the same family with the same molecular formula, they exhibited different effects towards lung cancer whereby beta-carotene gained its bad reputation in increasing lung cancer risk while lycopene did the opposite of it. It was proposed that lycopene could have a special role in lung cancer and it may have a selective ability towards lung cancer.

Though with the new perspectives towards the anti-cancer activity of lycopene, it is still insufficient for scientists to gauge the real potential of lycopene in cancer prevention. It is worth investigating whether the cancer suppression by inflammation resolution of lycopene could be a synergistic outcome of its own respective anti-inflammatory and anti-cancer activity. In terms of activation of the immune system to suppress cancer, it is crucial to fully understand the mechanism behind it and how the immunomodulatory effects of lycopene could set off the anti-cancer activity. The difference in terms of how beta-carotene could be harmful towards lung cancer and how lycopene could be selective towards lung cancer may rely on the difference between the chemical structures of both carotenoids and their distinct different metabolites. It is also worth investigating how both of the carotenoids could impact retinoic acid signaling pathway differently.

## Figures and Tables

**Table 1:** Summary of cell culture studies evaluating anti-cancer properties of lycopene.

Compound	Subject	Experiment Design	Outcome	Reference
Lycopene (Lyc)	SK-Hep-1 cells (highly invasive hepatoma cell line)	Treatment with 1, 2.5, 5, 10, 20 µmol/L Lyc	↓cell migration, invasion (bell-shaped manner) ↑nm23-H1 (bell-shaped manner) nm23-H1 and cell migration and invasion (-ve r)	[16]
		Treatment with 1–10 µM Lyc	↓cell invasion, MMP-9, NF-κB, Sp1, IGF-1R, ROS	[17]
		Treatment with 1, 2.5, 5, 10 µM Apo-8′-lycopenal (Lyc metabolite), 10 µM Lyc	(Lyc, Apo-8′-lyc)↓cell invasion, migration (Apo-8′-lyc) ↓MMP-2, -9, Rho GTPase, ERK/p38, PI3K-Akt ↑nm23-H1, TIMP-1,-2	[18]
		Treatment with Lyc (0.1–5 µM), induced with TGF-β	↓NOX4 mRNA, NOX, ROS, cell migration, invasion, adhesion activity, MMP-9, MMP-2	[19]
	H-Ras MCF10A, MDA-MB231 (highly aggressive breast cancer cell)	Treatment with Lyc	↓cell invasion, migration, proliferation ↓ERK, Akt	[20]
	HT-29 cells (human colon cancer cells)	Treatment with Lyc	↓cell invasion, MMP-7, phosphorylation of Akt, GSK-3β, ERK ½, AP-1, β-catenin ↑E-cadherin stabilization	[21]
	ER/PR^+^ MCF-7, HER2^+^ SK-BR-3, MDA-MB-468 cell lines	Treatment with Lyc (168 h)	inhibition of cell cycle progression G_0_/G_1_ ↑PARP cleavage, ERK1/2, p21, Bax ↓cyclin D1, Akt, mTOR ↔Bcl-xL	[23]
	HGC-27 cell lines	Incubated with various conc of Lyc for 24, 48 or 72 h	↑LC3-I, p-ERK	[63]
	Balb/c nude mice model	Injected with HGC-27 cells, fed with 20, 30, 60 mg/kg Lyc per d, oral	↓tumour weight	
	Lymphocytes from human blood	Incubation with 10, 20, 40 µM/mL Lyc, before and after X-irradiation at doses of 0.5, 1 and 2 Gy	↓DNA damage Note: Lyc administration after irradiation, no effect Note: Low doses are useful	[64]
	(Pancreatic cancer) PANC-1 cells	Treatment with 0.25, 0.5 µM for 24 h	↓ROS, NF-κB, cIAP1, cIAP2, survivin ↑caspase-3, Bax:Bcl-2	[24]
	Mouse epidermal cell line, JBG P+ (JB6 Cl 41-5a)	Pretreatment with Lyc for 5 days, incubation with TPA, with or without Lyc for 14 days	↓colony formation, (mRNA) KEAP1 ↑(mRNA) SOD1, GSR, GPX1, CAT, GCLC, GCLM, NQO-1, HMOX1, nuclear NRF2 localization, LC3, p62	[65]
	Mice	Subjected to DMBA (60 µg) dissolved in 0.2 mL topically on back, after 1 week, TPA (4 µg) twice a week for 32 weeks; Control group (1), 8 µmol Lyc/d since first week (2), 8 µmol Lyc/d from first wk to 4th week only (3), 8 µmol Lyc/d since fourth week (4), Acetone/d since fourth week (5): 32 weeks experiment	↓incidence rate, multiplicity of cutaneous papillomas, increased in epidermal thickness, invasion of benign papillomas, 8-OHdG, 4HNE ↑survival rate, GSH/GSSG ratio, SOD, GR, GPx, CAT, (mRNA) SOD1, GSR, GPX1, CAT, GCLC, GCLM, NQO-1, HMOX1 Note: lycopene was more effective as a pretreatment and during promotion phase of induced tumors.NRF2 was required for the effect of lycopene-induced prevention against tumor	
Apo-10′-lycopenoic acid, A-10-LA (Lyc metabolite)	NHBE cells (human bronchial epithelial cells), BEAS-2B-immortalized normal bronchial epithelial cells, A549 (non-small cell lung cancer cells)	Treatment with apo-10′-lycopenoic acid	↓cyclin E inhibition of cell cycle progression G(1)→S ↑p21, p27, RAR beta	[14]
	A/J mouse model	NNK injection (induction) and supplemented (10, 40, 120 mg/kg of A-10-LA	↓tumor multiplicity	
	BEAS-2B cells	Treatment with apo-10′-lycopenoic acid	↑NRF2, HO-1, NAD(P)H dehydrogenase (quinone 1), GSTs, GCL, GSH	[66]
	Human liver THLE-2, HuH7 cells	Treatment with apo-10′-lycopenoic acid	↑SIRT1, p21, apoptosis ↓cyclin D1	
	C57BI/6J mice	Supplementation with A-10-LA (10 mg/kg) for 24 wks, high fat diet, induced with diethylnitrosamine	↓tumor multiplicity, volume, incidence, caspase-1, TNF-α, IL-6, NF-κB p65, STAT3, Akt, cyclin D1 ↑SIRT1, PARP cleavage	[25]
Apo-8′-lycopenal (Lyc metabolite)	Human HepG2 cells	Treatment with 1, 5, 10 µM Apo-8′-lycopenal (Lyc metabolite), 10 µM Lyc	↓cell invasion, migration ↑NRF2, HO-1, NQO-1 ↓KEAP1	[15]

Note: ‘↑’ indicates increment; ‘↓’ indicates decrement; ‘↔’ indicates no change.

**Table 2:** Summary of animal studies evaluating anti-cancer properties of lycopene.

Compound	Subject	Experiment Design	Outcome	Reference
Lycopene (Lyc)	High mammary tumor strain of SHN virgin mice	Control (1), 5 × 10^−5^% Lyc (2), AIN-76TM diet	↓mammary tumor development, TYMS, serum FFA, prolactin	[67]
	Sprague Dawley rats	N-methylnitrosourea (intrarectal, 1 wk), followed by administration of Lyc (1), lutein (2), α-carotene (3), β-carotene (4), palm carotene (5), daily gavage (wk 2 and wk 5)	↓aberrant crypt foci development	[77]
	Male weanling rats	Induction of hepatocarcinogenesis by 6 × 100 mg/kg BW diethylnitrosamine (DEN)/100 mg/kg BW 2-nitropropane (2-NP), fed with 300 mg/kg β-carotene (1), canthaxanthin (2), astaxanthin (3), Lyc (4), 15,000 retinol equiv. excess vit A (5), 3-methycholanthrene (6) intraperitoneal, 3–4 wks	↔No., size of preneoplastic liver foci ↓size of GGT^+^, GST^+^ foci, Liver volume fraction occupied by foci Note: modulate P-450 2E1, not antioxidant properties	[70]
	Multiorgan carcinogenesis B6C3F1 mice model	Combined treatment with diethylnitrosamine (DEN), N-methyl-N-nitrosourea (MNU) and 1,2-dimethylhydrazine (DMH), Lyc + water: 25/50 ppm (1), Control (2), Lyc only: 25/50 ppm (3), 21 wks	↓incidences and multiplicities of lung adenomas and carcinomas Note: restricted to male, G1 with 50ppm Lyc ↔aberrant crypt loci, tumors in colon and kidney among groups	[80]
	F344/NSlc rats	2 mg/ 4 mg N-methylnitrourea x 3 per wk (3 wks), plain water (1), 17 ppm Lyc (2), diluted tomato juice with 17 ppm Lyc (3), diluted tomato juice with 3.4 ppm Lyc (4)	(3) ↓colon cancer incidence, but not in (2)	[78]
	Hepatocellular carcinoma (HCC)LEC rats	Diet containing 0.005% Lyc (1), 1% TJ-9: crude extracts of 7 herbs (2), control (3) administered from 6 wks age to 76 wks age	↔number, mean area and % area GST-P-+ focal lesions (liver, HCC); Note: TJ-9 had higher number of GST-P-+ lesion in HCC), AFP, cumulative survival rates ↓iron conc. in liver	[81]
	N-methyl-N’-nitrosoguanidine (MNNG) and saturated NaCl (S-NaCl) induced Male Wistar rats	N-methyl-N’-nitrosoguanidine (MNNG) + saturated NaCl (1), MNNG + S-NaCl + Lyc (2), Lyc (3), Control (4)	↓gastric carcinomas ↑GSH, GPx, GST, GR	[73]
		MNNG + S-NaCl (1), MNNG + S-NaCl + Sallylcysteine (SAC) (2), MNNG + S-NaCl + Lyc (3), MNNG + S-NaCl + SAC + Lyc (4), chemoprevention agents (5–7), Control (8)	↔GSH (stomach, erythrocytes), GPx (liver, erythrocytes), GPx activities (stomach), Bax, Bim ↑GSH (liver), GPx (stomach), GSH activities, GPx activities (liver, erythrocytes), caspase-8 ↓tumor burden, Bcl-2	[26]
	Resistant hepatocyte (RH) model of hepatocarcinogenesis Wistar rats	70 mg/kg BW lutein (1), Lyc (2), Control (3)	↑liver carotenoid conc. ↔incidence, total number, multiplicity of hepatocyte nodules ↓No., size, area of GST^+^ preneoplastic lesions, hepatic DNA strand breakage	[71]
	Colon carcinogenesis Sprague Dawley rat model	Induction by azoxymethane, followed by treatment with diallylsulfide (1), Lyc (2), theaflavin (3)	↓aberrant crypt foci, preneoplastic lesion, COX-2, iNOS	[60]
	Nude mice	Supplementation 2× per wk (12 wks), with 1, 20 mg/kg BW Lyc, 20 mg/kg BW β-carotene; starting wk 2, injection with SK-Hep-1 cells via tail vein	↓MMP-2, VEGF, tumor metastasis, mean no. of tumors, tumor cross-sectional area, PCNA, MMP-9 ↑nm23-H1	[22]
	Hepatocarcinogenesis in rat model	Injected with diethylnitrosamine (DEN) and fed with control diet or high fat diet (HFD) with or without Lyc or tomato extract	(HFD + Lyc) ↓no. of GST+ hepatic foci, PCNA, cyclin D1, ERKs, NF-κB ↔TNF-α, IL-1β, IL-12, CYP2E1 ↑HO-1, NRF2	[72]
	N-methyl-N′-nitrosoguanidine (MNNG) gastric cancer rat model	Control (1), 200 mg/kg BW MNNG + saturated NaCl (2), 200 mg/kg BW MNNG + saturated NaCl + 50 mg/kg BW Lyc (3) 200 mg/kg BW MNNG + saturated NaCl + 100 mg/kg BW Lyc (4) 200 mg/kg BW MNNG + saturated NaCl + 150 mg/kg BW Lyc (5)	↑SOD, CAT, GSH-Px, IL-2, IL-4, IL-10, TNF-α, IgG, IgA, IgM ↓MDA, IL-6	[61]
	BCO2-knockout and wild-type male mice	Lyc supplementation (100 mg/kg diet, 24 wks), induced by high fat diet	(BCO2-KO) ↑hepatic Lyc, miR-199a/b, miR214 ↓hepatocellular carcinoma incidence, multiplicity, ER(UPR), Met mRNA, β-catenin, mTORC1 (Wild type) ↓NF-κB p65, STAT3, IL-6, inflammatory foci	[62]
	(In vitro) OV-MZ-6 cells	Treatment with 2, 5 µM Lyc	↓ITGA5, pERK 1/2 ↔ITGB1, tERK, vimentin	[75]
	(In vivo) Ovarian cancer-bearing mice	Prevention Gp: Placebo (1), Lyc (2) Treatment Gp: Placebo (1), Lyc (2), Lyc + Taxol (3), Taxol + Platin (4), Platin (5), Lyc + Taxol + Platin (6) Concentration of Lyc: 0.75 mg/mL	(Lyc Prevention Gp) ↓metastatic load, Ki67, ITGA5B1, ITGA5, ILK, ITGB1, FAK, MMP-9, serum and ascites CA125, EMT markers in metastatic tissue (Note: MMP-9 restricted to metastatic tissue, not tumor tissue) ↔tumor load, serum and ascites MMP-9 (Lyc Treatment Gp) ↓tumor load, Ki67, ITGA5, ITGA5B1, ascites CA125 ↑MMP-9 ↔ILK, ITGB1, FAK, serum and ascites MMP-9, serum CA125	
	Laying hens	Control (1), 200 mg/kg per kg diet Lyc (2), 400 mg/kg per kg diet Lyc (3)	↓incidence, no. and size ovarian tumor, rate of adenocarcinoma, MDA, NF-κB, STAT3 ↑NFE2, HO-1	[76]
	(In vitro) Lewis lung carcinoma (LLC) cells	Control (1), Lyc: 10 µM (2), Lyc: 20 µM (3), Lyc: 40 µM (4)	↑(mRNA) IFNβ, IFNγ, IRF1, IRF7, CXCL9, CXCL10, pJAK, pSTAT3 ↓(mRNA) DMNT3a, methylation levels of promoters (IRF1, IRF7), PD-1 due to IFNγ, pAkt ↔(mRNA) DNMT1, DNMT3b	[69]
	(In vivo) C57BL/6 mice	Control (1), Anti PD-1, 6 mg/kg (2), Lyc, 40 mg/kg (3), Anti-PD-1 + Lyc (4); intraperitoneal, 3 days, 4 times	↓tumor volume, weight, IL-4, IL-10, (mRNA) DMNT3a, methylation levels of promoters (IRF1, IRF7) ↑IL-2, IFNγ, CD4^+^:CD8^+^, % IFNγ^+^/CD8^+^ T cell, % perforin^+^/CD8^+^ T cell, % granzyme B^+^/CD8^+^ T cell, (mRNA) IFNβ, IFNγ, IRF1, IRF7, CXCL9, CXCL10 ↔(mRNA) IRF3, IRF8, DNMT1, DNMT3b	
	CD-1 mice in AOM-DSS model	Normal (1), AOM+DSS control (2), (*Bifidobacterium longum*) BF + AOM + DSS (3), BF + Lyc 20 mg/kg + AOM + DSS (4), BF + Lyc 50 mg/kg + AOM + DSS (5), Lyc 20 mg/kg + AOM + DSS (6), Lyc 50 mg/kg + AOM + DSS (7), Metformin + AOM + DSS (8)	↑positive rates of IGF-1, IGF-2 (high dose), IGF-1R, IGF2BP1, IGFBP2 (low dose), IGFBP3 (high dose), lymphocyte infiltration ↓inflammation incidence, positive rates of IGF-2 (low dose), IGFBP2 (high dose), IGFBP3 (low dose) ↔No. of tumors, adenocarcinomas incidence Note: presence of focal necrosis	[79]
Lycopene-Enriched Tomato Oleoresin (LETO)	Rat mammary tumor model	Induced with 7, 12-dimethyl-benz[a]anthracene (DMBA) 2 wks, followed by injection of 10mg/kg LETO (1), β-carotene (2), control (3) twice per wk, 16 wks	↑plasma, hepatic Lyc ↓tumors, tumor area	[68]
		Supplementation with 250 ppm Lyc (1), 500 ppm (2), 250 ppm lycopene-rich tomato carotenoid oleoresin (TCO) (3), 500 ppm TCO (4), control (5) followed by initiation with N-methylnitrosourea (NMU) (7 days) 18 wks experimentation	↔tumor incidence, latency, multiplicity, volume, total tumors per group Note: supplementation with TCO ↑serum Lyc conc. > supplementation with pure Lyc	[82]

Note: ‘↑’ indicates increment; ‘↓’ indicates decrement; ‘↔’ indicates no change.

**Table 3:** Summary of clinical trials evaluating anti-cancer properties of lycopene.

Compound	Subject	Experiment Design	Outcome	Ref
Lycopene (Lyc)	47,894 human subjects initially free of diagnosed cancer	Validated semiquantitative food-frequency questionnaire	↓risk of non-stage A1 prostate cancer	[83]
	26 male patients with prostate cancer, 14 stage T1, 12 stage T2	Control (1), 15 mg Lyc (2)	↓plasma prostate-specific antigen (PSA) ↑connexin 43 ↔Bcl-2, Bax Note: sample size is relatively small	[86]
	47,365 participants	Dietary questionnaires	↓risk of prostate cancer Note: moderate association	[84]
	32 patients with localized prostate adenocarcinoma	Randomized placebo-controlled study: 30 mg Lyc/day	↑serum and prostate Lyc conc., apoptotic index (hyperplastic and neoplastic cells) ↓serum PSA, leukocyte 8OHdG	[87]
	58,279 men aged 55–69 yrs: 642 prostate cancer cases	Cohort study, 6.3 yrs follow-up, semi-quantitative food-frequency questionnaire	↔risk of prostate cancer	[96]
	69 men with favourable risk prostate cancer	3-month randomized, double blinded clinical trial: 30 mg/day Lyc (1), 3 g/day fish oil (2), placebo (3)	↔IGF-1, COX-2	[97]
	11 cohort studies, 6 nested case–control studies	Meta-analysis	OR < 1 (high tomato intake and incidence of prostate cancer) Modest effect in prevention of prostate cancer	[89]
	26 studies with 17,517 cases of prostate cancer, from 563,299 participants	Meta-analysis	↓risk of prostate cancer (Lyc: 9–21 mg/day; plasma Lyc: 2.17–85 µg/dL)	[90]
	18 prospective cohort studies in 2012	Pooled analysis (interval collapsing method)	Protective effect towards ER^−^/PR^+^ or ER^−^/PR^−^ breast cancer	[91]
Plasma Lycopene	25,802 persons: 103 men with prostate cancer, 103 men as control	Analysis of serum	↔risk of prostate cancer	[94]
	209 prostate cancer cases, 228 control, Black and white men in US (40–79 yrs old)	Analysis of serum carotenoids	↔risk of prostate cancer, only useful particularly for aggressive disease Note: insignificant inverse association (serum Lyc and prostate cancer)	[95]
	450 incident prostate cancer cases	Case-control study nested within prospective Health Professionals Follow-up Study	↓risk of prostate cancer Note: restricted to older participants, without family history	[85]
	521 women with breast cancer	Analysis of serum using HPLC	↓risk of breast cancer among premenopausal women and all ER/PR subtypes	[92]
	17 prospective studies with 3603 cases, 458,434 participants	Meta-analysis	Nonlinear dose-dependent (lung cancer and plasma Lyc) Note: stronger inverse association at low plasma Lyc conc.)	[93]
Lycopene-rich tomato	79 prostate cancer patients	Nutritional intervention: tomato products with 30 mg Lyc (1), tomato products + selenium, omega-3 fatty acids, soy isoflavones, grape/pomegranate juice and green/black tea (2), Control (3)	↓PSA level	[88]

Note: ‘↑’ indicates increment; ‘↓’ indicates decrement; ‘↔’ indicates no change.

## Abbreviations

SR-B1	Scavenger receptor class B type 1
CD36	Cluster of differentiation 36
ISX	Intestine-specific homeobox
BCO1	β-carotene oxygenase 1
IOM	Institute of Medicine
GRAS	Generally recognized as safe
MHC II	Major histocompatibility complex II
IL-2	Interleukin-2
MAPK	Mitogen-activated protein kinase
ERK	Extracellular signal-regulated kinases
JNK	c-Jun N-terminal kinases
NF-κB	nuclear factor kappa-light-chain-enhance of activated B cells
LPS	Lipopolysaccharides
ICAM-1	Intercellular adhesion molecule 1
VCAM-1	Vascular cell adhesion molecule 1
HUVEC	Human umbilical vein endothelial cell
TNF-α	Tumor necrosis factor-alpha
ONOO^−^	Peroxynitrite
MDA	Malondialdehyde
NO	Nitric oxide
ROS	Reactive oxygen species
Bax	Bcl-2-associated X protein
Bcl-2	B-cell lymphoma 2
NRF2	Nuclear factor erythroid 2-related factor 2
ARE	Antioxidant response element
p-IκB	Phosphorylation of IκB
NFE2L2	Nuclear factor erythroid derived 2-like 2
HMOX1	Heme-oxygenase 1 gene
NQO-1	NAD(P)H dehydrogenase [quinone] 1
c-AMP	Cyclic adenosine monophosphate
MMPs	Matrix metalloproteinases
iNOS	Inducible nitric oxide synthase
COX-2	Cyclooxygenase-2
PGE2	Prostaglandin E2
Lyc	Lycopene
ET-1	Endothelin 1
MCP-1	Monocyte chemoattractant protein-1
RelA	Nuclear factor NF-κB p65 subunit
MIP-2	Macrophage Inflammatory Protein 2
DSS	Dextran sulfate sodium
TUNEL	Terminal deoxynucleotidyl transferase dUTP nick end labeling
EGFP	Enhanced green fluorescent protein
AST	Aspartate Aminotransferase
ALT	Alanine aminotransferase
LDH	Lactate dehydrogenase;
GGT	Gamma-glutamyl transferase
RAGE	Receptor for advanced glycation endproducts
eWAT	epididymal white adipose tissue
ATM	Adipose tissue macrophage
STAT6	Signal transducer and activator of transcription 6
Akt	Protein kinase B
LDL	Low-density lipoprotein
CRP	C-reactive protein
CAT	Catalase
SOD	Superoxide dismutase
GPx	Glutathione peroxidase 1
TGF-β1	Transforming growth factor beta 1
Cyt c	Cytochrome c
GSH	Glutathione
HO-1	Heme-oxygenase 1
GCLC	Glutamate-cysteine ligase catalytic subunit
BSA	Bovine serum albumin
IgG	Immunoglobulin G
CFA	Complete Freund’s adjuvant
SAPK	Stress-activated kinases
BW	Body weight
CYP2E1	Cytochrome P450 2E1
IFNγ	Interferon γ
MPO	Myeloperoxidase
HC	Hyperhomocysteinemic control group
ICV	Intracerebroventricular
L-NAME	L-NG-Nitro arginine methyl ester
SAH	Subarachnoid hemorrhage
RAI	Radioactive iodine
DM	Diabetes mellitus
CP/CPPS	Chronic prostatitis/chronic pelvic pain syndrome
TLE	Tomato lycopene extract
Th1	Th1 helper cells
TLR	Toll-like receptor
NAD(P)H	Reduced form of nicotinamide adenine dinucleotide phosphate
4HNE	4-hydroxynonenal
RAR	retinoic acid receptor
ATRA	all-trans retinoic acid
RARE	retinoic acid response elements
